# Walking, Cycling and Driving to Work in the English and Welsh 2011 Census: Trends, Socio-Economic Patterning and Relevance to Travel Behaviour in General

**DOI:** 10.1371/journal.pone.0071790

**Published:** 2013-08-21

**Authors:** Anna Goodman

**Affiliations:** Faculty of Epidemiology and Population Health, London School of Hygiene and Tropical Medicine, Keppel Street, London, United Kingdom; Old Dominion University, United States of America

## Abstract

**Objectives:**

Increasing walking and cycling, and reducing motorised transport, are health and environmental priorities. This paper examines levels and trends in the use of different commute modes in England and Wales, both overall and with respect to small-area deprivation. It also investigates whether commute modal share can serve as a proxy for travel behaviour more generally.

**Methods:**

23.7 million adult commuters reported their usual main mode of travelling to work in the 2011 census in England and Wales; similar data were available for 1971–2001. Indices of Multiple Deprivation were used to characterise socio-economic patterning. The National Travel Survey (2002–2010) was used to examine correlations between commute modal share and modal share of total travel time. These correlations were calculated across 150 non-overlapping populations defined by region, year band and income.

**Results:**

Among commuters in 2011, 67.1% used private motorised transport as their usual main commute mode (−1.8 percentage-point change since 2001); 17.8% used public transport (+1.8% change); 10.9% walked (−0.1% change); and 3.1% cycled (+0.1% change). Walking and, to a marginal extent, cycling were more common among those from deprived areas, but these gradients had flattened over the previous decade to the point of having essentially disappeared for cycling. In the National Travel Survey, commute modal share and total modal share were reasonably highly correlated for private motorised transport (r = 0.94), public transport (r = 0.96), walking (r = 0.88 excluding London) and cycling (r = 0.77).

**Conclusions:**

England and Wales remain car-dependent, but the trends are slightly more encouraging. Unlike many health behaviours, it is more common for socio-economically disadvantaged groups to commute using physically active modes. This association is, however, weakening and may soon reverse for cycling. At a population level, commute modal share provides a reasonable proxy for broader travel patterns, enhancing the value of the census in characterising background trends and evaluating interventions.

## Introduction

In recent years, promoting walking and cycling for transport (‘active travel’) has moved up multiple policy agendas, including in relation to health, transport and climate change. Active travel provides one route whereby people can integrate moderate-to-vigorous intensity physical activity into their everyday lives [1]–[3], and participating in active travel is independently associated with a wide range of health benefits [4]–[7]. Active travel is also more likely than recreational physical activity to displace journeys by cars [8], which in turn is expected to reduce noise, congestion, road traffic crashes, urban air pollution and the emission of greenhouse gases [9]–[11].

Despite these potential benefits, levels of walking and cycling declined in the second half of the twentieth century in Britain, while motorised transport increased [12]. The past two decades have, however, seen some hints that these trends may be at least partially reversing. The UK is one of various high-income countries in which levels of car use have flattened or slightly declined, as have the proportion of adults holding a driving licence [13]–[15]. Simultaneously, much greater policy focus has been given to promoting and investing in active travel, often particularly in relation to cycling [3], [16]–[21]. In London, successive Mayors have launched initiatives both to encourage cycling (e.g. a bicycle sharing system) and to discourage driving (e.g. the introduction of a ‘congestion charge’ for cars entering central London). Nationally, initiatives have included the publication of an Active Travel Bill in Wales and an Active Travel Strategy for the UK [19], [21]; the allocation of £1 billion to local sustainable transport initiatives; and the implementation of town-wide initiatives in 18 ‘cycling towns’ [22]. Such interventions may explain the upward trend in cycling reported in London [23] and in the original six cycling towns [22]. The first aim of this paper is to contextualise these setting-specific findings using newly-released census 2011 data. Specifically, I aim to examine national and regional levels and trends of walking, cycling and driving to work in England and Wales.

A second aim is to examine changes in the distribution of these different commute modes with respect to small-area deprivation. In 2010, the Strategic Review of Health Inequalities in England called for research to monitor the social gradient of active travel [24]. This call was prompted by data in the original six ‘cycling towns’ indicating that higher social grade was associated with a higher probability of reporting any past-week cycling [22]. Similarly in London, higher household income is positively associated with making at least one trip by bicycle on any given day [25], while higher area affluence is positively associated with using the bicycle sharing system [26]. In a previous analysis of census data from 1971–2001, individuals from lower social classes were more likely to walk or cycle to work but this effect became less strong over time [27]. This paper examines whether this trend has continued, and therefore whether changes in commuting patterns might tend towards widening health inequalities.

The final aim of this paper is methodological. The UK census is publically available and provides a uniquely large and representative source of information, with very high geographical resolution. It therefore provides one potentially powerful means of examining trends in travel behaviour and/or evaluating the impact of interventions, particularly those made at a sub-regional or local level. The census is, however, severely limited in including only one question on travel behaviour, namely ‘usual main commute mode’. By contrast, most research studies and policy evaluations are more interested in total travel behaviour. The value of the census data therefore depends considerably on how far it can be used as a proxy for total travel behaviour, at least at the population level. This paper uses National Travel Survey data to examine this issue, as well as to contextualise the census data in other ways.

## Methods

### Census Data on main Commute Mode in England and Wales

The British census happens every ten years and is compulsory for all residents. In England and Wales, the estimated proportion of people covered by the census was 96% in 1991, 94% in 2001 and 94% in 2011 [28], [29] This paper takes the 2011 census as its starting point (data available from www.ons.gov.uk/ons/guide-method/census/2011/index.html) and makes comparisons with previous censuses (data available from http://casweb.mimas.ac.uk). Ethical approval was not required as all data are fully in the public domain.

For all respondents aged 16–74 with a current job, the 2001 and 2011 census data includes responses to the question “How do you usually travel to work? (Tick one box only, tick the box for the longest part, by distance, of your usual journey to work)”. This data is also available for a 10% random sample of the 1971, 1981 and 1991 censuses (see File S1 for details of minor differences in the 1971 and 1981 response options). I categorised responses into five commute modes: cycling; walking; public transport; private motorised transport (car, van or motorcycle, as a driver or passenger); and other modes. I calculated the modal share of each of these modes as a proportion of all commuters, i.e. excluding people not in work or people working at or from home. All adults reporting that their home address was also their place of work were treated as non-commuters. Note that this final decision was necessary to allow comparable analyses across the censuses, but differs from some previous stand-alone analyses of census 2011 data ([30], see File S1 for details).

### Small-area Deprivation, Adjusting for Geographical Remoteness

The 2010 English Index of Multiple Deprivation (IMD) [31] is a weighted composite of small-area data relating to seven deprivation domains, assigned at the level of lower super output areas (LSOA, average population around 1500). There is also a 2011 Welsh IMD [32], but differences in the constituent domains and variables mean that the two scores are not directly comparable. I therefore created hundredths of deprivation separately in England and Wales and combined these into a single variable capturing each LSOA’s ranking within its country.

The standard IMD score includes a small number of indicators capturing distance to services (e.g. distance to the nearest post office). This complicates interpretations of associations with commute mode, since these indicators may serve as a straightforward proxy for average commute distance. I therefore created an ‘IMD-minus-distance to services’ score, employing an approach that has been used elsewhere to remove particular domains from the overall score [33], [34] (see File S1 for details). All substantive findings were unchanged in sensitivity analyses which used only the income deprivation domain.

To adjust for geographical remoteness in the equity analyses, I used rankings on the IMD ‘distance to services’ subdomain. I also used the 2004 Rural and Urban Area Classification [35] to assign settlement type (three-level categorical variable: urban area with a population >10,000; smaller towns and fringe areas; and villages, hamlets and isolated dwellings); and to assign sparseness (binary variable denoting whether the LSOA was in the bottom 5% for population density in the surrounding 30 km).

### The National Travel Survey

The National Travel Survey is a continuous, population-based survey of households in Britain (annual sample size around 8100 households in recent years, household participation rate around 60% [36]). This paper uses National Travel Survey data for fully-participating adults (aged 16 years or over) from 2002 to 2010 (available from http://www.esds.ac.uk). All members of participating households complete questionnaires, which cover the usual main commute mode for all working participants. These questionnaires are also used to create fifths of real household income equivalised for household composition [37]. All participants additionally complete one-week travel diaries that include the time taken and distance travelled for all stages of most trips. Motor vehicle trips off the road network are excluded (e.g. on private land), as are walking and cycling trips where the surface is unpaved or access is restricted (e.g. on private land, across open countryside or in a park that is closed at night) [38].

I first used data from trip stages in the National Travel Survey to examine what proportion of total travel time in each mode was captured directly by the question on ‘main mode to work’. For example, I calculated what proportion of the total time spent cycling by adults was accounted for by commute trips made by individuals who reported cycling as their usual main mode. I then created 150 non-overlapping subpopulations within the National Travel Survey based on 10 regions (9 standard English regions plus Wales), three time periods (2002–2004, 2005–2007 and 2008–2010) and the five income fifths (10*3*5 = 150). For each subpopulation, I calculated the proportion of participants reporting each mode as their usual main method of travelling to work (‘commute modal share’). I also used data from trip stages to calculate the proportion of total travel time spent in each mode (‘total modal share’). This allowed me to examine how far the commuting data available in the census predicted the more general outcome of ‘total travel’.

### Statistical Analyses

Most analyses rely on the presentation of raw percentages (plus binomial proportion confidence intervals) or raw Pearson correlation coefficients. When analysing the National Travel Survey data, I calculated commute modal share and total modal share for each subpopulation using the household-, individual- and trip-level weights provided. These weights adjust for factors such as differential non-response rates by age, sex and region, and for the fact that participants only reported short walks (<1 mile) on the final day [36]. I then present raw correlation coefficients between commute and total modal share for these 150 subpopulations. The results were very similar if each subpopulation was weighted for its population size (mean 830 commuting adults, range 231–1765).

For the equity analyses using census data, I fitted linear regression models with commute modal share as the outcome (e.g. proportion commuting by bicycle) and with twentieth of small-area deprivation as the main predictor variable. LSOAs were the unit of analysis, and I accounted for spatial autocorrelation by fitting two-level random intercept models of LSOAs nested within local authorities (equation in File S1). I adjusted these models for settlement type, sparseness and IMD ‘distance to services’ rank, entering the former two as categorical variables and the latter one using linear plus quadratic terms. I used Stata 12 for all statistical analyses, and used ArcGIS 10.1 to create maps.

## Results

### National Levels and Trends in the 2011 Census

**Figure 1:**
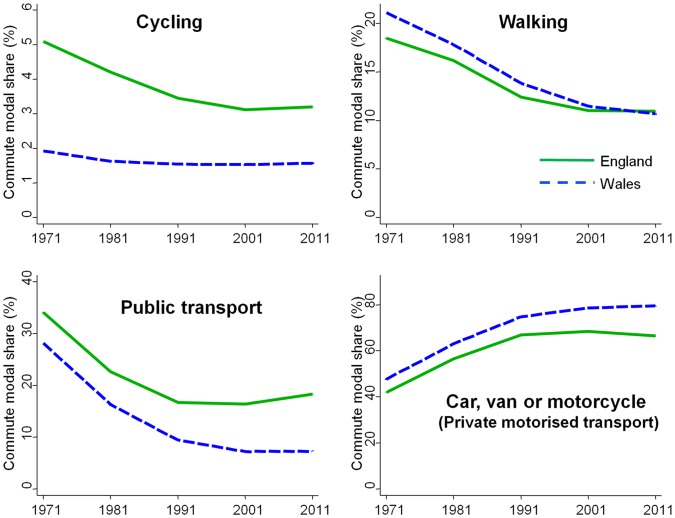
National levels and trends in usual main commute modes across the English and Welsh censuses 1971–2011. Data tabulated in Table S1 in File S2, along with subcategories of motorised modes and 95% confidence intervals. Confidence intervals are not presented here as they are too narrow to be visible.

41.1 million adults aged 16–74 took part in the 2011 English and Welsh census, of whom 14.6 were not in employment, 2.8 million worked at or from home, and 23.7 million commuted to work. Table 1 presents the distribution of their usual main commute modes, while Figure 1 compares these to the previous four censuses. Commute modal share was dominated by private motorised transport: cars, vans or motorcycles represented the usual main mode of 67.1% of commuters (66.4% in England, 79.4% in Wales). This was followed by public transport (17.8%) and walking (10.9%), and finally by cycling (3.1%) and ‘other’ modes (1.1%).

**Table 1:** Modal share of usual main commute modes among commuters in the 2011 English and Welsh censuses, and change since 2011.

	England (N = 22,484,295)		Wales (N = 1,213,283)		England & Wales (N = 23,697,578)	
	Modal share 2011	Change since 2001	Modal share 2011	Change since 2001	Modal share 2011	Change since2001
**Cycling**	3.20%	0.09%	1.57%	0.04%	3.11%	0.08%
**Walking**	10.93%	−0.07%	10.66%	−0.80%	10.91%	−0.11%
**Public transport**	18.33%	1.92%	7.32%	0.07%	17.77%	1.82%
**Bus**	8.17%	−0.10%	5.05%	−0.77%	8.01%	−0.14%
**Train or underground**	10.16%	2.02%	2.27%	0.84%	9.76%	1.95%
**Car, van or motorcycle**	66.46%	−1.94%	79.43%	0.82%	67.13%	−1.78%
**Car or van driver**	60.11%	−0.34%	71.41%	3.58%	60.69%	−0.13%
**Car or van passenger**	5.46%	−1.26%	7.41%	−2.54%	5.56%	−1.32%
**Motorcycle**	0.89%	−0.33%	0.61%	−0.22%	0.87%	−0.33%
**Taxi or other**	1.08%	0.00%	1.02%	−0.14%	1.08%	−0.01%

See Table S1 in File S2 for confidence intervals and for modal share tabulated since 1971. All changes between 2001 and 2011 are significant (all p<0.05, almost all p<0.001) except the change in ‘taxi or other’ commuting in England.

Although most commuters still reported using private motorised transport, the trends suggested that this mode might be reaching saturation and perhaps starting to decline. In England, the decade between 2001 and 2011 saw a modest decrease in private motorised transport (−1.9 percentage points) and a concomitant increase in public transport (+1.9%, with this effect being driven by an increase in train commuting: see Table 1). Although these changes are relatively small in absolute terms, they acquire some additional importance when considered in light of the longer-term trends in the opposite directions (Figure 1). In this context, even the marginal changes in walking (−0.07%) and cycling (+0.09%) are somewhat encouraging when compared to the comparatively large declines in previous decades. As for Wales, it differed from England in that private motorised transport continued to increase and walking showed a more marked decrease. These changes occurred at a slower rate than in previous decades, however, suggesting that in future years these trends may stabilise or even reverse in Wales (as already appears the case for public transport).

Finally, it is worth noting that decreases in private motorised transport were largely or entirely confined to commuting as a car/van passenger or by motorcycle (Table 1). Driving oneself to work by car or van (which accounted for the vast majority of private motorised transport) showed only a very small decrease in England (−0.3%) and a notable increase in Wales (+3.6%). This suggests that changes in the proportion of commuters putting cars on the road (and therefore contributing to congestion, air pollution and road traffic crashes) have been less favourable than the changes in overall private motorised commuting presented in Figure 1.

### Regional Levels and Trends in the 2011 Census

**Figure 2:**
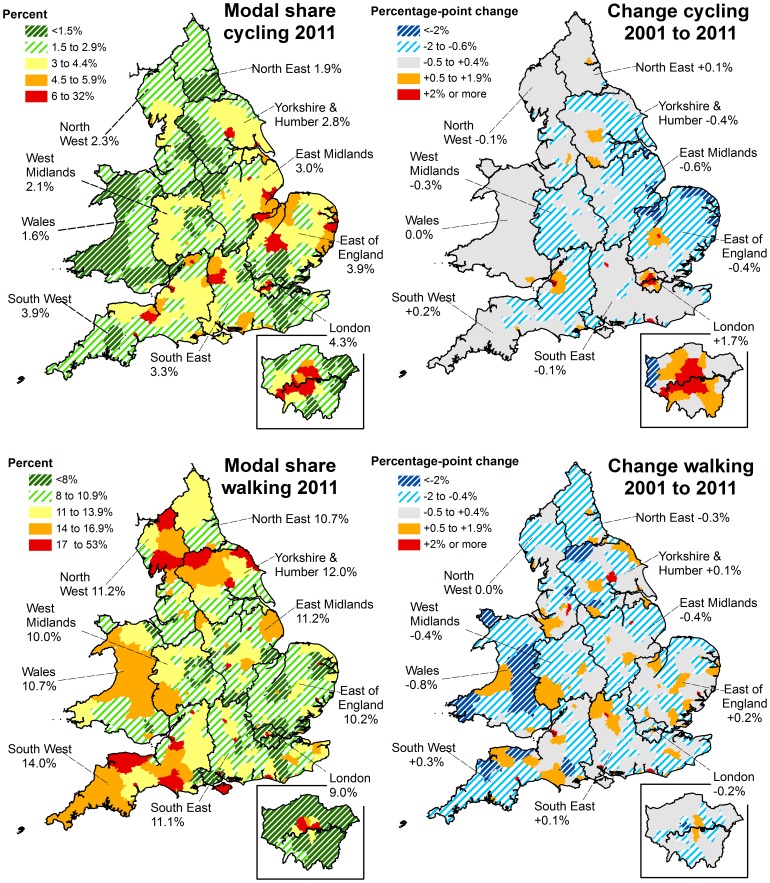
Regional levels and trends in walking and cycling to work, 2001 and 2011 census. The left panels present the proportion of commuters using a) cycling (top half) and b) walking (bottom half) as their usual main commute mode, in England and Wales in 2011. The right panels present the change in these modal shares (2011 minus 2001). Local authorities are the units of analysis, but averages are presented for each region. See Figure S1 in File S2 for equivalent maps for public transport and private motorised transport, and File S3 for all 2001 and 2011 modal shares tabulated by local authority.

Figure 2 shows how these overall levels and trends in cycling and walking to work varied across England and Wales, and also which areas showed the greatest increases relative to 2001. For cycling, London stood out as the only region to have experienced a marked increase (+1.7%, versus −0.6% to +0.2% in all other regions), an increase largely concentrated in inner London. This led London to overtake the East of England as the region with the highest cycle commute modal share. For walking there was less variation at the regional level, both in absolute walking levels and in the change since 2001. At a local level, the highest levels of walking and cycling (both 60%) were in the only two local authorities with a commuting population under 5000 (the Isles of Scilly and the City of London). Apart from these, the local authorities with the highest levels of cycling were the university towns of Cambridge (32.6%, 4.2 percentage point increase from 2001) and Oxford (19.1%, 2.8% increase) and the London borough of Hackney (15.4%, 8.5% increase). Bristol also stood out alongside London as a large city (population 430,000) which had substantially increased its modal share (8.2%, 3.3% increase from 2001). The local authorities with the highest levels of walking were the small, historic cities of Norwich (24.8%, 0.5% increase from 2001) and Exeter (24.1%, 3.8% increase).

Equivalent maps for travel by private motorised transport and public transport are presented in Figure S1 in File S2, while File S3 tabulates all 2001 and 2011 commute modal shares for all local authorities. At a regional level, London was an outlier, with much higher levels of commuting by public transport than other regions (53%, vs. 7–14% elsewhere) and much lower levels of private motorised transport (32%, vs. 71–79% elsewhere). This difference was more pronounced in 2011 than a decade previously, reflecting the fact that London had showed the largest regional increases in public transport commuting since 2001 (+7.3%) and the largest decreases in private motorised transport (−8.8%). Yet while these changes were largest in London, the other southern regions also showed increases in public transport (+0.2 to +2.0%) and decreases in private motorised transport (−0.6 to −1.9%). By contrast the opposite was generally true in the Midlands, Wales and the North of England. At the local authority level, the highest levels of commuting by private motorised transport were all in rural areas, with the highest proportion in East Dorset (88.5%, 0.8% increase since 2001).

### Equity Analyses: Socio-economic Distribution of Commute Modes

**Figure 3:**
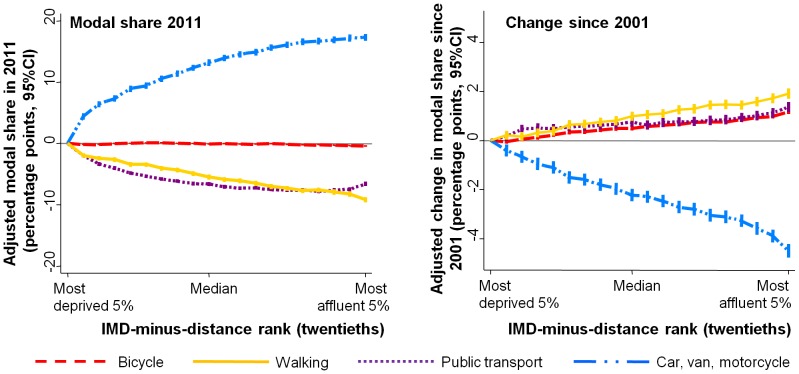
Associations between small-area deprivation and levels and trends in commute mode, 2001 and 2011 censuses. The left panel presents associations with main commute mode in 2011 (England and Wales combined), the right panel presents associations with change in usual main commure mode (2011 minus 2001). Analyses are from eight separate multi-level linear regression models, adjusting for three measures of geographical remoteness and using Lower Super Output Areas as the unit of analysis (population around 1500). Deprivation is measured in country-specific twentieths.

Across the entire gradient for small-area deprivation, greater affluence was associated with a higher proportion of commuters using cars, vans or motorcycles as their main mode (see Figure S2 in File S4 for raw data, see the left panel of Figure 3 for multi-level models adjusting for geographical remoteness). Simultaneously, greater affluence was progressively associated with a lower proportion of commuters walking or (except for a slight reversal in the very most affluent areas) using public transport. For example, the raw proportion of commuters using walking as their main mode was 6.7% in the most affluent tenth versus 15.4% in the most deprived tenth, translating into an adjusted difference of −7.5 percentage points (95%CI −8.0, −7.0). Cycling was fairly equal across the socio-economic gradient but was also slightly more common in deprived areas, with an adjusted difference of −0.60% (95%CI −0.77, −0.44) between the most affluent versus the most deprived tenth. Very similar patterns of commute modal share were seen across fifths of household income in the National Travel Survey in 2008–2010, the only notable exception being a more marked increase in public transport commuting among the most affluent income fifth (see Figure S2 in File S4). This broad similarity suggests that the associations observed in the census with respect to small-area deprivation may also apply with respect to individual-level measures of socio-economic position.

Although greater affluence predicted lower walking, public transport use and cycling in the 2011 census, this was less true than it had been a decade earlier. As shown in the right-hand panel of Figure 3, increasing affluence progressively predicted an increase in these three modes between 2001 and 2011, and a decrease in private motorised transport. These findings were unchanged when using earlier IMD versions, all of which were highly correlated (e.g. r = 0.98 between the 2004 and 2010 versions).

**Figure 4:**
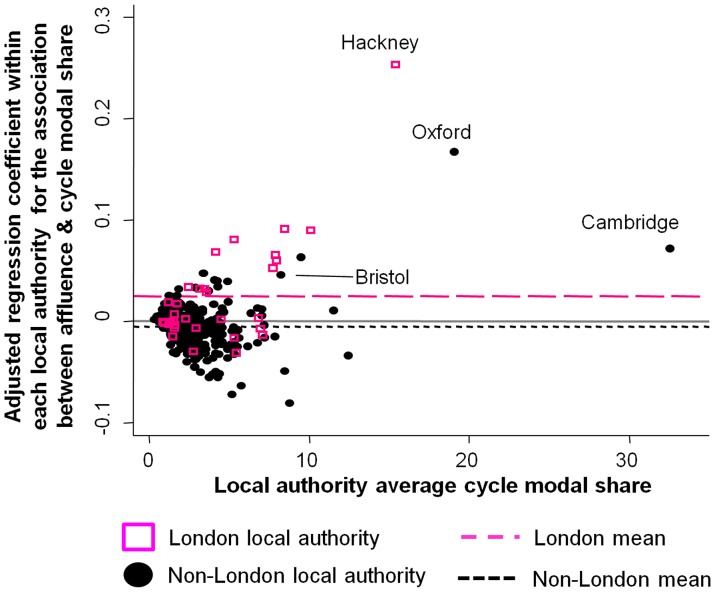
Association between cycle modal share in each local authority and the within-local-authority relationship between affluence and cycle commuting. The x-axis presents the cycle modal share in each local authority in the 2011 census (England and Wales combined). The y-axis presents regression coefficients capturing the percentage increase in commute modal share within that local authority (2011 minus 2001) for each percentile increase in affluence, adjusting for three measures of geographical remoteness. These regression analyses were conducted for each local authority separately (N = 346), using Lower Super Output Areas as the unit of analysis (population around 1500). Two very small local authorities (N<5000 commuters) were excluded.

The pattern of findings was very similar when analysing England and Wales separately, and these gradients were also generally apparent within local authorities. For example, the average within-local-authority association between commute mode and affluence was significantly positive for private motorised transport, significantly negative for walking and public transport, and marginally-significantly negative for cycling (see Table S2 in File S4). For cycling, however, Cambridge, Oxford and Hackney were notable exceptions and showed strong positive associations between greater affluence and greater cycle commuting (see Figure 4). Similarly, Greater London was the only region of England or Wales where the average within-local-authority gradient was significantly positive, and there was also a modest positive gradient in Bristol (the largest city to have experienced a substantial cycling increase). Thus not only had the negative socio-economic gradient for cycling flattened over time, but it was inverted in England’s highest-cycling areas and in its highest-cycling region.

### Setting the Census Findings in Context: Data from the National Travel Survey

Thus far, this paper has made comparisons across years, across regions and across socio-economic groups with respect to the only travel data available in the census, namely usual main mode for commuting to work. This final section uses National Travel Survey data to examine how these findings can be expected to reflect differences in travel behaviour more widely. A useful starting point is to consider what proportion of total travel time in each mode is directly captured by the census. Among adult participants in 2008–2010, 31% of all cycling time was reported during commute trips by individuals who stated that cycling was their ‘usual main commute mode’. A further 10% of all cycling time was reported during commute trips made by adults who gave a different usual main mode, i.e. capturing people who used cycling as part of a multi-modal trip or who cycled only occasionally. The remaining 59% of all cycling time was reported during non-commute trips (this includes any cycling by adults not in employment).

The cycling picked up by the census question therefore corresponds to around a third of total adult cycling time. This proportion was similar for public transport (30% vs. 4% during other commute trips and 66% during non-commute trips), but was lower for car use (20% vs. 2% and 78%) and very low for walking (6% vs. 8% and 86%). Indeed, slightly less time was reported walking in commute trips where walking was the usual main mode than was reported during other commute trips (6% vs. 8%). Two-thirds of this ‘other commute’ walking were accounted for by multimodal public transport trips. Both here and for the analyses reported below, these findings were very similar when using travel distance instead of time.

**Figure 5:**
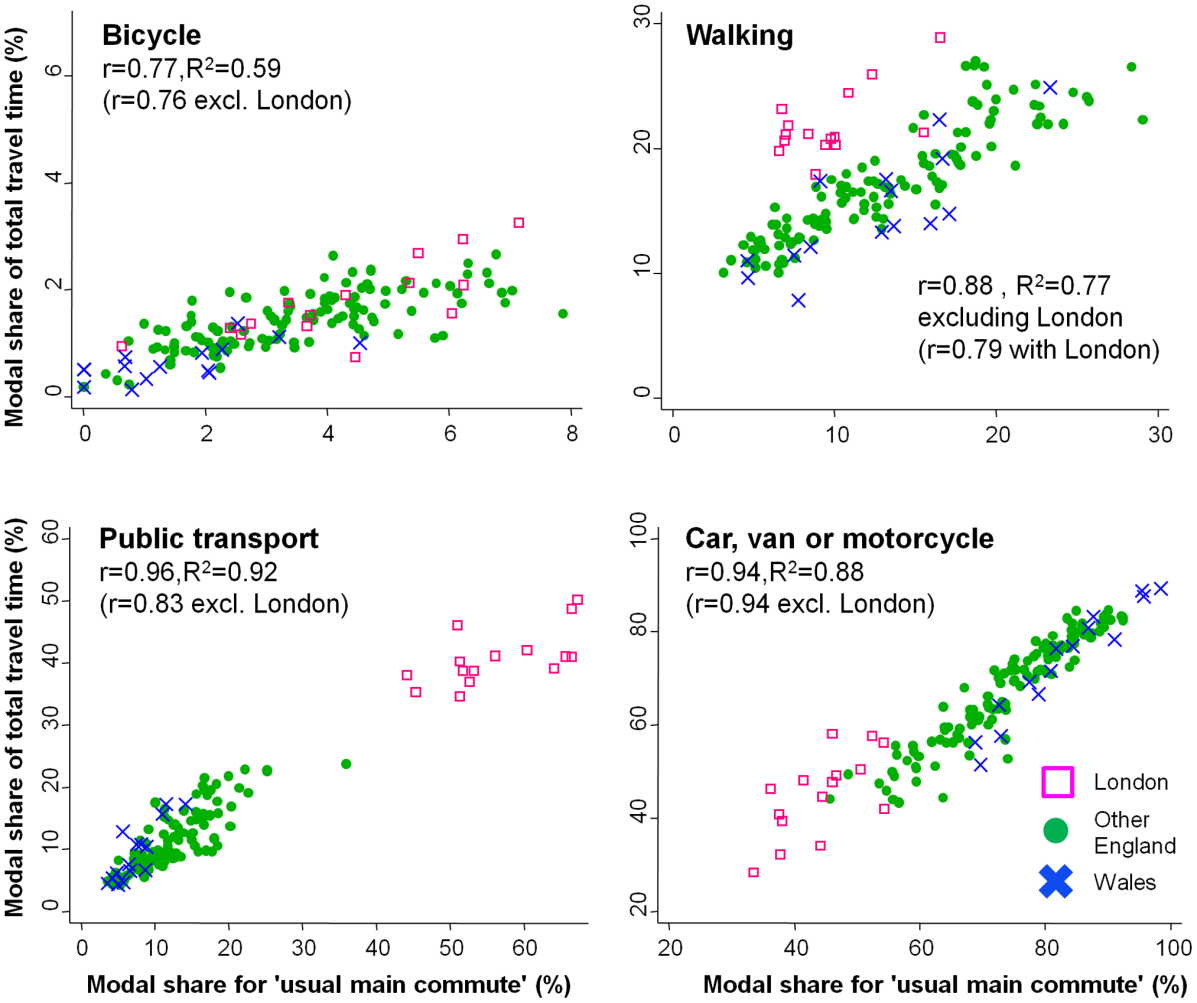
Associations between commute modal share and total modal share, National Travel Surveys 2002–2010. These panels present raw associations between commute modal share (based on usual main commute mode) and modal share of total travel time in 150 non-overlapping populations. These populations were defined by region, year band and income fifth, using data from the National Travel Surveys 2002–2010.

Although capturing only a minority of total travel time, the census question served as a reasonably good proxy measure for total modal share at the population level. This is indicated in Figure 5, which presents correlation coefficients of 0.77–0.96 between the commute modal share and the proportion of total travel time spent in that mode. These correspond to R^2^ values of 0.59–0.92, i.e. across these 150 populations commute modal share explained between 59% and 92% of the variance in total modal share. Visual inspection indicated that populations defined by region, year band or income all seemed to share broadly the same distribution (see Figures S3 and S4 in File S5). The only major exception was that high levels of public transport meant that total walking levels were higher than expected in London, hence the decision to highlight correlation coefficients excluding London in Figure 5.

Interestingly, over the observed range of commute modal shares for public transport and car use, the line of best fit of the scatter graphs in Figure 5 was reasonably similar to the line of identity (i.e. intercept zero, gradient one: see Table S3 in File S5 for equations for lines of best fit). In other words, if 20% of a population used public transport as their usual main commute mode, that population also spent approximately 20% of its total travel time in public transport. By contrast, for cycling and walking the lines of best fit differed more markedly from the line of identity. Instead a given commute modal share predicted a smaller share of total travel time for cycling and a larger share for walking.

### Relative versus Absolute Measures of Travel Time

It is important to remember that the findings presented in the previous section all relate to modal share, i.e. the relative proportion of travel by different modes. A final contribution of National Travel Survey data is to caution that such relative differences do not necessarily correspond to equivalent absolute differences, because populations may differ in their absolute trip rates or travel time. This is not a major issue for the regional and temporal comparisons because average daily travel times showed relatively little variation across regions (e.g. ranging from 55–65 min across all regions in 2008–2010, except in London where it is up to 69 min) or over time (e.g. ranging from 64 min in 2002–2004 to 62 min in 2008–2010). It is, however, very important for the socio-economic comparisons because total travel time showed a strong dose-response association with income. For example, total daily travel time ranged from an average of 51 min/day among adults living in the lowest income fifth in 2008–2010 to 59 min/day in the middle fifth and 77 min/day in the highest fifth.

As a result, although the *proportion* of active travel time was greatest in low income groups (24%, 18% and 15% among the lowest, middle and highest fifths), absolute active travel time showed much less difference (15 min/day, 12 min/day and 13 min/day among the lowest, middle and highest fifths: see Table S4 in File S5 for analyses treating walking and cycling separately). Conversely, the association between high income and percentage travel time in private motorised modes became even larger when converted into absolute travel times (50%, 67% and 70% for the proportion of travel time among the lowest, middle and highest fifths; 25 min, 39 min and 53 min for absolute daily travel time). A similar point can be made in relation to the 2011 census. Although this paper always uses ‘all commuters’ as a denominator, one could instead use ‘total adult population’ if one wanted to focus on absolute volumes of commuting travel. Given that the proportion of adults in employment was higher in more affluent areas (e.g. 70% vs. 54% in the most vs. least affluent fifth), using this alternative denominator would attenuate the socio-economic gradient in active commute modes and strengthen the gradient in private motorised transport.

## Discussion

The 2011 census indicates that private motorised transport continues to dominate commuting in England and Wales, representing 67% of usual main commute modes. This contrasts with modal shares of 18% for public transport, 11% for walking, and 3% for cycling. Somewhat more encouragingly, the long-term increase in private motorised commuting has halted across England and Wales as a whole (and even shown a small decline), while public transport, walking and cycling have risen or remained relatively stable for the first time in decades. With respect to socio-economic position, higher affluence continues to predict a lower commute modal share of walking and, to a marginal extent, cycling. Nevertheless these negative gradients have flattened over time and the gradient for cycling is reversed in the highest cycling locations. Because affluent individuals travel more in total, these socio-economic associations with commute modal share cannot be assumed to correspond directly to associations with absolute travel times or distances. Nevertheless, commute modal share does generally appear to be a reasonably good proxy measure (at the population level) for the *relative* proportion of travel time spent in different modes.

### Strengths and Limitations

In interpreting these findings, it is important to consider this paper’s strengths and limitations. A key strength is the integration of data from complementary sources. The census represents a national sample with a uniquely high response rate, and therefore maximises power and generalisability. By contrast, alternative data sources such as the National Travel Survey or London Travel Demand Surveys have smaller sample sizes (18,000–19,000 individuals in 2009/10) and more potential for participation bias (response rates 52–60%) [36], [39]. These other datasets do provide much richer travel information, however, hence my use of National Travel Survey data to contextualise and partially overcome some of the census’s limitations.

The greatest limitation of the census is that it only covers travel for commuting and only covers the ‘usual main mode of travel’ for these commute trips. As I demonstrate using National Travel Survey data, this only captures a minority of total travel time (6–31%, depending on mode). Even when considering only commute journeys, the census question captures less than half of all commute walking time and only three-quarters of all commute cycling time. Although I demonstrate that commute modal share generally provides a reasonable proxy for total modal share, this may not be true in settings with distinctive transport characteristics (e.g. a high density of park-and-ride facilities, and therefore many multi-modal commute journeys).

Another limitation is that the British census is only conducted every ten years, and therefore cannot be used to examine the precise timing of changes in commuting patterns. In addition, the census 2011 data is currently only available at the small-area level, meaning I could only present equity analyses with respect to area deprivation. Reassuringly, this showed a broadly similar pattern to individual-level analyses of income in the National Travel Survey. Nevertheless, other socio-economic indicators may show different patterns of association [40], and multiple indicators may be needed to characterise fully the socio-economic structure of commuting [41]. It would therefore be valuable to complement the equity analyses presented here with an examination of individual socio-economic and demographic predictors of commute modal share, once samples of individual anonymised records are released. In addition, individual-level analyses could build upon this paper by examining who is changing their travel behaviour, for example whether middle-aged men show the largest increases in cycling, as suggested by previous national surveys [27], [42]. Future analyses could also explore associations with geographic factors such as hilliness, climate and land use patterns; although outside the scope of this paper, these may play a key role in explaining local and regional variation [43].

### Implications of Levels and Trends in Different Commute Modes

This paper adds to the evidence that, after increasing for decades, levels of car use in England and Wales may now flattening or declining [13]–[15]. If so, this suggests that forecasts by the Department for Transport may overestimate future demand for car travel by assuming that this demand will continue to increase [14]. This finding also offers some hope for the prospect of creating a more physically active and less environmentally polluting transport system. Nevertheless, the 2011 census underlines the scale of the challenge faced in achieving this, with two-thirds of the working population currently using cars, vans or motorcycles as their usual main commute mode.

An equivalently mixed picture is offered with respect to cycling levels. On the one hand, even the very small national increase in cycling is something to celebrate when compared to previous decades of decline. London in particular stands out as a region that has achieved an impressive increase in its cycle commute modal share over the past decade. Nevertheless cycling continues to be very rare in most parts of England and Wales, and so is not realising its potential to confer substantial health and environmental benefits [44], [45]. Among other things, this suggests that the examination of relative inequalities in this and other reports should not distract from the fact that cycling is (too) rare (and driving (too) common) in all socio-economic groups. Similarly, although cycling often gets more attention from policy-makers and academics, these census data serve as a reminder that walking is a far more common source of active commuting. This is particularly the case given the evidence in this and previous [46] reports indicating the large volume of walking accumulated during multimodal public transport trips.

### Implications of the Socio-economic Patterning of Different Modes

This paper confirms previous research indicating that motorised transport (and associated carbon emissions) are higher among socio-economically advantaged groups [47]–[49]. This may be relevant when considering the best policy options to shift to a low-carbon transport economy. For example, it might be that fuel or parking charges would have to rise considerably to have a substantial effect upon travel demand in these more affluent groups [47], and that effective and equitable policies would also need to include other measures (e.g. increasing the supply of attractive alternative commute options) [49].

By contrast, the modal share for active commuting was lower in more affluent areas, thereby contrasting with many other health behaviours such as smoking, poor diet or leisure-time physical activity [40], [50]–[52]. This at first seems at odds with the concern raised in the recent Strategic Review of Health Inequalities in England that differential participation in active travel might tend to widen health inequalities [24]. One reason for this difference is that the Strategic Review focussed on cycling, which showed a much flatter socio-economic gradient in the census. This therefore again highlights the importance of considering walking when looking at population-level sources of active travel, and particularly when considering more deprived areas [53]. Secondly, some previous studies have considered recreational as well as transport walking and cycling [22], [26]; these two may have different correlates, with the former being more likely to show a positive association with affluence [40]. Thirdly, some previous evaluations have focussed on locations like Cambridge (e.g. [41]), which this paper shows to be atypical in having a higher cycle modal share among people from more affluent areas.

A final, key factor is that the focus in this paper has been the relative measure of ‘modal share’, whereas previous studies have examined absolute measures such as ‘total travel time’ or ‘any participation’ [22], [25], [26]. As demonstrated in this paper, these two approaches may generate qualitatively different associations with socio-economic position, and research in this field therefore needs to distinguish clearly between travel modal share and total travel volume. A focus on modal shares is likely to be more meaningful for some research questions, for example when evaluating the impacts of interventions targeting modal choice. From a broader perspective, however, it is important not to lose sight of socio-economic differences in total travel time. Ignoring these differences may risk overstating the physical activity benefits accruing to the poor or understating the harms generated by motorised transport among the rich.

Creating an equitable transport system therefore needs to focus not only on equalising access to different modes, but also on equalising access to the potential for travel in general [49]. This is arguably particularly important given that the 2011 census suggests a continuation of the trend for the modal shares of walking and cycling to increase more rapidly among socio-economically advantaged groups [27]. Moreover, those areas that have successfully attained or maintained a high cycling modal share are also precisely the areas where cycling is most concentrated among the affluent. These two findings suggest that in the future cycling may become increasingly concentrated among more affluent groups, both in terms of modal share and, to an even greater extent, in terms of time spent cycling. To the extent that transport policies accelerate or diffuse these and other trends outlined in this paper, they may widen or narrow inequalities with respect to a range health and social outcomes [54], [55].

### Methodological Implications

Besides highlighting the need to distinguish between relative and absolute measures of travel, this paper makes a methodological contribution through examining correlations between commute modal share and total modal share. It is important to stress that this paper only examines the strength of these associations at the population level. At the individual level the associations may be weaker, particularly as the individual-level determinants of modal choice for commuting often differ in important ways from those governing other journey purposes (e.g. [56], [57]).

Nevertheless, except when comparing walking levels between London and elsewhere, population-level commute modal share does appear a reasonable proxy for the proportion of total travel time that adults in that population spend in that mode. This suggests that the census data can cautiously be used as an indicator for travel behaviour in general, which in turn enhances their value for evaluating transport interventions implemented at the local or regional level. This paper therefore highlights the potential power of the census not only to characterise ‘the state of the nation’, but also to evaluate attempts to shift that ‘state’ to one which is better for public health and the environment.

## Supporting Information

File S1 **Further details on methods.**
(DOC)

File S2 **Tabulation of results and additional analyses: national and regional trends.** This file contains Table S1 and Figure S1. Table S1, Modal share of usual main commute modes among commuters in England and Wales (percent and 95% confidence interval). Figure S1, Regional levels and trends in taking a) public transport and b) private motorised transport to work, 2001 and 2011 census.
(DOC)

File S3 **Tabulation of modal shares in 2001 and 2011, for all local authorities in England and Wales.**
(XLS)

File S4 **Additional analyses: equity.** This file contains Table S2 and Figure S2. Table S2, Average adjusted change in commute modal share per percentile increase in affluence, in 346 local authorities of England and Wales. Figure S2, Comparison of commute modal share a) in the census by small area deprivation and b) in the National Travel Survey by equivalised household income.
(DOC)

File S5 **Additional analyses: data from the National Travel Survey.** This file contains Table S3, Table S4, Figure S3, and Figure S4. Table S3, Parameters of lines of best fit (univariable regression) between commute modal share (x variable) and share of total travel time (y variable). Table S4, Distribution across fifths of equivalised household income of a) the relative proportion of total travel time in different modes and b) the absolute average daily travel time in different modes: data from the National Travel Survey 2008–2010. Figure S3, Association between commute modal share and modal share of total travel time in 150 populations defined by region, year band and income fifth, distinguishing sub-populations by year. Figure S4, Association between commute modal share and modal share of total travel time in 150 populations defined by region, year band and income fifth, distinguishing sub-populations by income.
(DOC)
