# Supplementary material for: Walking, Cycling and Driving to Work in the English and Welsh 2011 Census: Trends, Socio-Economic Patterning and Relevance to Travel Behaviour in General
Source: PLoS One. 2013 Aug 21;8(8):e71790. doi: 10.1371/journal.pone.0071790 (PMC3749195; doi:10.1371/journal.pone.0071790)
Supplement: File S1 — Further details on methods. (DOC) [file pone.0071790.s001.doc]

S1) Further details on methods

**Minor differences in the 1971 and 1981 census response options, and change in the definition of working-from-home between 2001 and 2011**

The 1971 and 1981 censuses both asked respondents for their usual main commute mode to work, but differed slightly from later censuses in the response options provided. The 1971 and 1981 censuses did not disaggregate 'other' commute modes from 'missing' data, so it was assumed that 0.5% of trips were made by other modes in England and Wales (the 1991 value). In addition the 1971 census combined walking with working at home. Extrapolating from the trend between 1981 and 2001, it was assumed that 3% of employees in England in 1971 worked from home and 5% in Wales.

Another change took place between 2001 and 2011 in the treatment of people who 1) recorded their home address as their main place of work but 2) also chose a commute method such as ‘driving a car’. This could, for example, happen if someone worked from home, but used a car to visit clients. In the 1971-2001 censuses, these people were automatically re-assigned to the commute mode ‘working at or from home’. By contrast, in 2011 the primary release of the census data left these people classified as ‘driving a car’ . This was considered useful for transport planning purposes, but undermines the comparability of the census over time and arguably also does not strictly capture ‘commuting’ travel. In this paper I therefore used the ‘alternative’ release of the census 2011 data which, as in previous decades, re-assigns those people to be ‘working at or from home’ (census table CT0015EW, and commissioned table CT0050).

**Calculation of an ‘IMD-minus-distance to services’ score**

As when calculating the full Index of Multiple Deprivation (IMD) score , I standardized and exponentially transformed all individual domain scores. I then calculated new weights by reallocating the 4.7% weight of the ‘distance to services’ subdomain across the other domains, in proportion to their original weights. I did the same for the Welsh IMD, reallocating the 10% weight of the ‘geographical access to services’ score across the other domains.

Note that these results are based on the Lower Super Output Areas (LSOAs) defined in the 2001 census, as it is for these LSOAs that IMD scores are available. The 2011 reported data in terms of LSOAs which had in some cases undergone minor modifications to their boundaries. Specifically, 97.5% were identical to their 2001 counterparts; 1.1% had been subdivided from a single 2001 LSOA; 0.9% had been merged from one or more 2001 LSOAs; and 0.5% had undergone other changes. For the 98.6% of LSOAs which were identical or had been subdivided, it was straightforward to convert these back to their 2001 equivalents. The remaining 1.4% were converted back under the assumption that each constituent 2001 LSOA had contributed equally to the 2011 LSOA. Excluding these problematic LSOAs had no effect on any findings.

**Multilevel random intercepts model used to examine the gradient in commute modes by area deprivation**

For my equity analyses, I fitted linear regression models with commute modal share as the outcome (e.g. proportion commuting by bicycle) and with twentieth of small-area deprivation, settlement type, sparseness and tenth of IMD ‘distance to services’ entered categorically as predictor variables. These regression models accounted for spatial autocorrelation by fitting two-level random intercept models, of LSOAs (the unit of analysis) nested within local authorities:

Y_ij_ = β _0_ + β_1_x_1ij_+...+β_p_x_pij_ + LA_j_  + e_ij_

Where Y_ij_ is the proportion commuting by a given mode within the *i*th LSOA in the *j*th local authority; β_1_...β_p_ are the parameters for the fixed effects of interest (x_1ij..._x_pij_), for example twentieths of area deprivation; LA_j_ is a random intercept for commute mode in the *i*th LSOA; and e_ij_ is the residual error term. Random intercepts were assumed to be normally distributed, allowing different variance parameters for each random intercept and for the residual error, and were estimated using maximum likelihood estimation. As a sensitivity analysis, I repeated these analyses with logged commute modal shares, in recognition of the fact that these proportions were often somewhat skewed. This did not change the substantive findings, and I therefore present analyses using untransformed outcomes to make the results easier to interpret.

**References**

1. Leveson Gower T (2013) 2011 census analysis - method of travel to work in England and Wales report. London: Office for National Statistics.

2. DCLG (2011) The English Indices of Deprivation 2010. London: Department for Communities and Local Government
