# Supplementary material for: Walking, Cycling and Driving to Work in the English and Welsh 2011 Census: Trends, Socio-Economic Patterning and Relevance to Travel Behaviour in General
Source: PLoS One. 2013 Aug 21;8(8):e71790. doi: 10.1371/journal.pone.0071790 (PMC3749195; doi:10.1371/journal.pone.0071790)
Supplement: File S2 — Tabulation of results and additional analyses: national and regional trends. This file contains Table S1 and Figure S1. Table S1, Modal share of usual main commute modes among commuters in England and Wales (percent and 95% confidence interval). Figure S1, Regional levels and trends in taking a) public transport and b) private motorised transport to work, 2001 and 2011 census. (DOC) [file pone.0071790.s002.doc]

**S1) Tabulation of results and additional analyses: national and regional trends**

Table S1: Modal share of usual main commute modes among commuters in England and Wales (percent and 95% confidence interval)

|  |  | **Modal share 1971** | **Modal share 1981** | **Modal share 1991** | **Modal share 2001** | **Modal share 2011** | **Change from 2001 to 2011** |
| --- | --- | --- | --- | --- | --- | --- | --- |
| **England** | **Cycling** | 5.09 (5.06, 5.12) | 4.20 (4.18, 4.23) | 3.45 (3.42, 3.47) | 3.11 (3.11, 3.12) | 3.20 (3.19, 3.21) | 0.09 (0.08, 0.09) |
|  | **Walking** | 18.44 (18.39, 18.50) | 16.14 (16.08, 16.19) | 12.38 (12.33, 12.43) | 11.00 (10.98, 11.01) | 10.93 (10.91, 10.94) | -0.07 (-0.08, -0.06) |
|  | **Public transport** | 34.08 (34.01, 34.15) | 22.67 (22.61, 22.73) | 16.73 (16.68, 16.78) | 16.41 (16.39, 16.42) | 18.33 (18.31, 18.35) | 1.92 (1.91, 1.94) |
|  | **Bus** | 26.37 (26.31, 26.43) | 16.12 (16.07, 16.18) | 9.96 (9.92, 10.01) | 8.27 (8.26, 8.28) | 8.17 (8.16, 8.18) | -0.10 (-0.11, -0.09) |
|  | **Train/**  **underground** | 7.71 (7.67, 7.75) | 6.55 (6.51, 6.58) | 6.77 (6.73, 6.80) | 8.14 (8.13, 8.15) | 10.16 (10.15, 10.17) | 2.02 (2.01, 2.04) |
|  | **Car, van or motorcycle** | 41.88 (41.81, 41.96) | 56.49 (56.42, 56.56) | 66.96 (66.89, 67.02) | 68.40 (68.38, 68.42) | 66.46 (66.44, 66.48) | -1.94 (-1.96, -1.92) |
|  | **Car/van**  **driver** | Not asked | Not asked | 57.48 (57.41, 57.55) | 60.45 (60.43, 60.47) | 60.11 (60.09, 60.13) | -0.34 (-0.36, -0.32) |
|  | **Car/van**  **passenger** | Not asked | Not asked | 7.81 (7.77, 7.85) | 6.72 (6.71, 6.73) | 5.46 (5.45, 5.47) | -1.26 (-1.27, -1.25) |
|  | **Motorcycle** | 1.78 (1.76, 1.80) | 3.16 (3.13, 3.18) | 1.67 (1.65, 1.68) | 1.22 (1.22, 1.23) | 0.89 (0.89, 0.89) | -0.33 (-0.34, -0.33) |
|  | **Taxi or other** | 0.50 (0.49, 0.51) | 0.50 (0.49, 0.51) | 0.49 (0.48, 0.50) | 1.08 (1.08, 1.09) | 1.08 (1.08, 1.09) | 0.00 (0.00, 0.00) |
| **Wales** | **Cycling** | 1.93 (1.84, 2.02) | 1.62 (1.54, 1.70) | 1.55 (1.47, 1.62) | 1.53 (1.51, 1.55) | 1.57 (1.55, 1.59) | 0.04 (0.02, 0.06) |
|  | **Walking** | 21.10 (20.84, 21.36) | 17.80 (17.56, 18.04) | 13.82 (13.61, 14.04) | 11.46 (11.40, 11.52) | 10.66 (10.61, 10.72) | -0.80 (-0.85, -0.74) |
|  | **Public transport** | 28.12 (27.84, 28.41) | 16.31 (16.08, 16.54) | 9.42 (9.24, 9.61) | 7.25 (7.20, 7.30) | 7.32 (7.27, 7.37) | 0.07 (0.02, 0.12) |
|  | **Bus** | 26.70 (26.42, 26.98) | 15.04 (14.82, 15.27) | 8.09 (7.93, 8.26) | 5.82 (5.78, 5.86) | 5.05 (5.01, 5.09) | -0.77 (-0.81, -0.73) |
|  | **Train/**  **underground** | 1.42 (1.34, 1.49) | 1.27 (1.20, 1.34) | 1.33 (1.26, 1.40) | 1.43 (1.41, 1.45) | 2.27 (2.24, 2.29) | 0.84 (0.81, 0.86) |
|  | **Car, van or motorcycle** | 47.64 (47.33, 47.96) | 63.07 (62.76, 63.37) | 74.69 (74.42, 74.96) | 78.60 (78.52, 78.68) | 79.43 (79.35, 79.50) | 0.82 (0.75, 0.90) |
|  | **Car/van**  **driver** | Not asked | Not asked | 61.72 (61.41, 62.02) | 67.83 (67.74, 67.91) | 71.41 (71.33, 71.49) | 3.58 (3.50, 3.66) |
|  | **Car/van**  **passenger** | Not asked | Not asked | 11.67 (11.47, 11.87) | 9.95 (9.89, 10.00) | 7.41 (7.36, 7.45) | -2.54 (-2.59, -2.49) |
|  | **Motorcycle** | 1.21 (1.15, 1.28) | 2.35 (2.25, 2.44) | 1.31 (1.24, 1.38) | 0.83 (0.81, 0.85) | 0.61 (0.60, 0.63) | -0.22 (-0.23, -0.20) |
|  | **Taxi or other** | 1.20 (1.13, 1.27) | 1.20 (1.13, 1.27) | 0.51 (0.47, 0.56) | 1.16 (1.14, 1.18) | 1.02 (1.00, 1.04) | -0.14 (-0.15, -0.12) |
| **England & Wales** | **Cycling** | 4.94 (4.91, 4.97) | 4.08 (4.05, 4.10) | 3.35 (3.33, 3.38) | 3.03 (3.03, 3.04) | 3.11 (3.11, 3.12) | 0.08 (0.07, 0.09) |
| **combined** | **Walking** | 18.54 (18.48, 18.59) | 16.22 (16.17, 16.28) | 12.45 (12.41, 12.50) | 11.02 (11.01, 11.03) | 10.91 (10.90, 10.92) | -0.11 (-0.12, -0.10) |
|  | **Public transport** | 33.81 (33.75, 33.88) | 22.36 (22.30, 22.42) | 16.36 (16.31, 16.42) | 15.95 (15.93, 15.97) | 17.77 (17.75, 17.78) | 1.82 (1.80, 1.83) |
|  | **Bus** | 26.41 (26.35, 26.47) | 16.08 (16.02, 16.13) | 9.87 (9.83, 9.91) | 8.14 (8.13, 8.16) | 8.01 (8.00, 8.02) | -0.14 (-0.15, -0.13) |
|  | **Train/**  **underground** | 7.40 (7.36, 7.44) | 6.29 (6.25, 6.32) | 6.49 (6.46, 6.53) | 7.80 (7.79, 7.82) | 9.76 (9.75, 9.77) | 1.95 (1.94, 1.97) |
|  | **Car, van or motorcycle** | 42.21 (42.14, 42.28) | 56.84 (56.77, 56.91) | 67.34 (67.28, 67.41) | 68.91 (68.89, 68.93) | 67.13 (67.11, 67.15) | -1.78 (-1.80, -1.76) |
|  | **Car/van**  **driver** | Not asked | Not asked | 57.69 (57.62, 57.76) | 60.82 (60.80, 60.84) | 60.69 (60.67, 60.71) | -0.13 (-0.15, -0.11) |
|  | **Car/van**  **passenger** | Not asked | Not asked | 8.00 (7.96, 8.04) | 6.88 (6.87, 6.90) | 5.56 (5.55, 5.57) | -1.32 (-1.33, -1.31) |
|  | **Motorcycle** | 1.76 (1.74, 1.77) | 3.12 (3.09, 3.14) | 1.65 (1.63, 1.67) | 1.20 (1.20, 1.21) | 0.87 (0.87, 0.88) | -0.33 (-0.33, -0.33) |
|  | **Taxi or other** | 0.50 (0.49, 0.51) | 0.50 (0.49, 0.51) | 0.49 (0.48, 0.50) | 1.09 (1.08, 1.09) | 1.08 (1.08, 1.08) | -0.01 (-0.01, 0.00) |

Figure S1: Regional levels and trends in taking a) public transport and b) private motorised transport to work, 2001 and 2011 census

The left panels present the proportion of commuters using a) public transport (top half) and b) car, van or motorcycle (bottom half) as their usual main commute mode, in England and Wales in 2011. The right panels present the change in these modal shares (2011 minus 2001). Local authorities are the units of analysis, but averages are presented for each region. See File S3 for all 2001 and 2011 modal shares tabulated by local authority.
