# Supplementary material for: Walking, Cycling and Driving to Work in the English and Welsh 2011 Census: Trends, Socio-Economic Patterning and Relevance to Travel Behaviour in General
Source: PLoS One. 2013 Aug 21;8(8):e71790. doi: 10.1371/journal.pone.0071790 (PMC3749195; doi:10.1371/journal.pone.0071790)
Supplement: File S4 — Additional analyses: equity. This file contains Table S2 and Figure S2. Table S2, Average adjusted change in commute modal share per percentile increase in affluence, in 346 local authorities of England and Wales. Figure S2, Comparison of commute modal share a) in the census by small area deprivation and b) in the National Travel Survey by equivalised household income. (DOC) [file pone.0071790.s004.doc]

**S4) Additional analyses: equity**

Figure S1: Comparison of commute modal share a) in the census by small area deprivation and b) in the National Travel Survey by equivalised household income


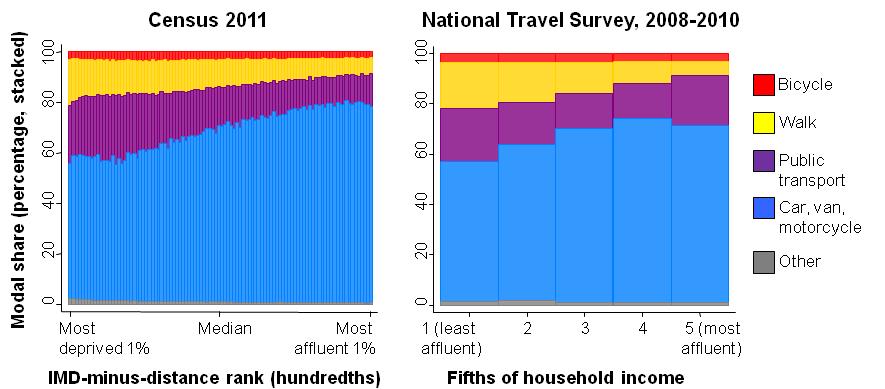


IMD=indices of multiple deprivation

Greater affluence was associated in the 2011 census with a much higher proportion of commuters using cars as their main mode, and a lower proportion using public transport or walking (Figure S1). There was relatively little gradient for cycling. In the multi-level models presented in the main text, adjusting for spatial correlation increased the negative gradient somewhat in the case of cycling. This increase reflected the fact that the within-region gradient in cycling levels was partly masked by higher absolute levels in some richer regions like the East of England. Simultaneously, however, the gradients for all modes were attenuated somewhat after adjusting for the three measures of geographical remoteness. This attenuation reflected the fact that many of the most deprived areas were inner-city areas where people might have only short distances to travel to work. Nevertheless, even in these adjusted models there remained strong evidence that higher deprivation predicted higher levels of walking and public transport in a roughly monotonic fashion, and correspondingly predicted lower levels of private motorised transport (Figure 3 in main text). These gradients were also generally seen at the local authority level (Table S1).

Table S1: Average adjusted change in commute modal share per percentile increase in affluence, in 346 local authorities of England and Wales

| **Commute mode** | **Regression coefficient (95%CI)** |
| --- | --- |
| **Cycling** | -0.003 (-0.006, 0.00005) |
| **Walking** | -0.093 (-0.100, -0.086) |
| **Public transport** | -0.033 (-0.042, -0.025) |
| **Private motorised transport** | 0.136 (0.124, 0.147) |

Regression coefficients represent the percentage increase in commute modal share for each percentile increase in affluence, adjusting for three measures of geographical remoteness. These regression analyses were conducted for each local authority separately (N=346), using Lower Super Output Areas as the units of analyses, and then averaged across local authorities for presentation in the Table. Two very small local authorities (N<5000 commuters) were excluded.
