# Supplementary material for: Walking, Cycling and Driving to Work in the English and Welsh 2011 Census: Trends, Socio-Economic Patterning and Relevance to Travel Behaviour in General
Source: PLoS One. 2013 Aug 21;8(8):e71790. doi: 10.1371/journal.pone.0071790 (PMC3749195; doi:10.1371/journal.pone.0071790)
Supplement: File S5 — Additional analyses: data from the National Travel Survey. This file contains Table S3, Table S4, Figure S3, and Figure S4. Table S3, Parameters of lines of best fit (univariable regression) between commute modal share (x variable) and share of total travel time (y variable). Table S4, Distribution across fifths of equivalised household income of a) the relative proportion of total travel time in different modes and b) the absolute average daily travel time in different modes: data from the National Travel Survey 2008–2010. Figure S3, Association between commute modal share and modal share of total travel time in 150 populations defined by region, year band and income fifth, distinguishing sub-populations by year. Figure S4, Association between commute modal share and modal share of total travel time in 150 populations defined by region, year band and income fifth, distinguishing sub-populations by income. (DOC) [file pone.0071790.s005.doc]

**S5) Additional analyses: data from the National Travel Survey**

Table S1: Parameters of lines of best fit (univariable regression) between commute modal share (x variable) and share of total travel time (y variable)

| **Mode** | **Sub-population** | **Beta regression coefficient, 95% CI** | **Intercept (alpha), 95% CI** | **Line of best fit** | **Range of observed commute modal share**† |
| --- | --- | --- | --- | --- | --- |
| Bicycle | All | 0.28 (0.24, 0.32) | 0.5 (0.4, 0.7) | y = 0.28x + 0.5 | 0 – 6 % |
| Walking | Non-London | 0.67 (0.61, 0.73) | 8.2 (7.4, 9.1) | y= 0.67x + 8.2 | 3 – 29% |
|  | London | 0.53 (0.11, 0.96) | 16.7 (12.4, 21.1) | y= 0.53x + 16.7 | 7 – 17% |
| Public transport | All | 0.84 (0.80, 0.88) | 2.1 (1.4, 2.8) | y= 0.84x + 2.1 | 3 – 54% |
| Car, van or motorcycle | All | 0.89 (0.83, 0.96) | 4.5 (-0.3, 9.2) | y= 0.89x + 4.5 | 32 – 89% |

†i.e. approximate range where these equations are most likely to be valid

Figure S1: Association between commute modal share and modal share of total travel time in 150 populations defined by region, year band and income fifth, distinguishing sub-populations by year


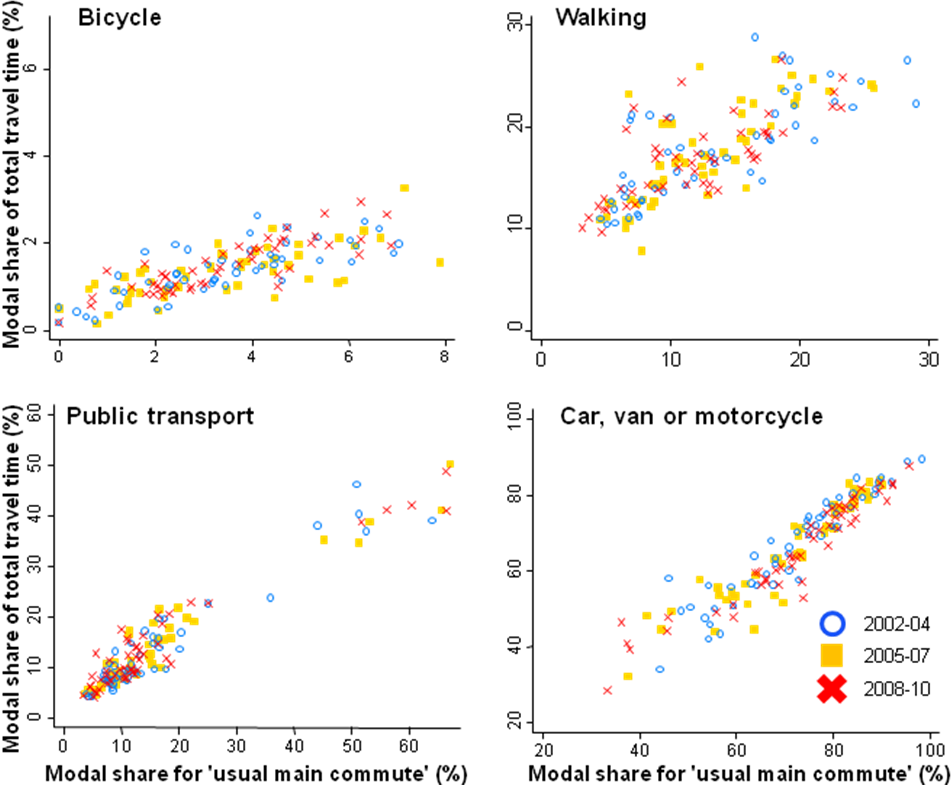


These panels present raw associations between commute modal share (based on usual main commute mode) and modal share of total travel time in 150 non-overlapping populations defined by region, year band and income fifth. They are based on data from the National Travel Surveys 2002-2010.

Figure S2: Association between commute modal share and modal share of total travel time in 150 populations defined by region, year band and income fifth, distinguishing sub-populations by income


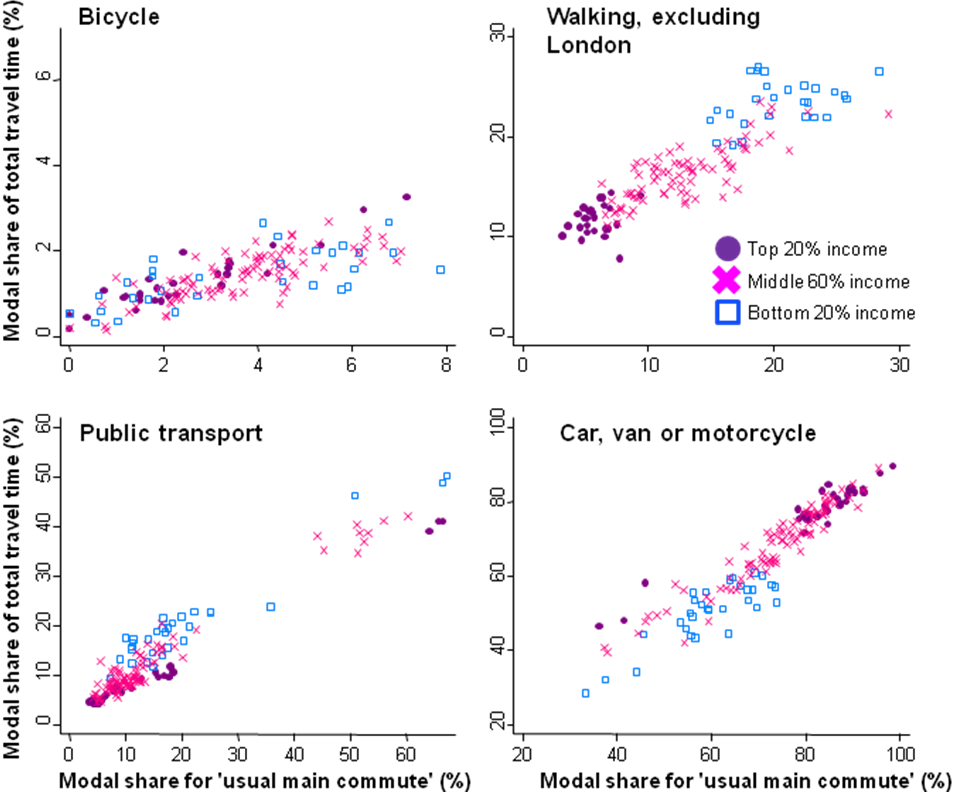


These panels present raw associations between commute modal share (based on usual main commute mode) and modal share of total travel time in 150 non-overlapping populations defined by region, year band and income fifth. They are based on data from the National Travel Surveys 2002-2010.

Table S2: Distribution across fifths of equivalised household income of a) the relative proportion of total travel time in different modes and b) the absolute average daily travel time in different modes: data from the National Travel Survey 2008-2010

| **Fifth of household** | **Cycling** | | **Walking** | | **Public transport** | | **Car, van or motorcycle** | | **Total daily** |
| --- | --- | --- | --- | --- | --- | --- | --- | --- | --- |
| **income** | **% travel time (95%CI)** | **Daily minutes (95%CI)** | **% travel time (95%CI)** | **Daily minutes (95%CI)** | **% travel time (95%CI)** | **Daily minutes (95%CI)** | **% travel time (95%CI)** | **Daily minutes (95%CI)** | **minutes (95%CI)** |
| **1 (lowest)** | 1.50 (1.24, 1.76) | 0.86 (0.69, 1.04) | 22.4 (21.5, 23.3) | 13.8 (13.1, 14.5) | 22.2 (21.2, 23.3) | 12.9 (12.1, 13.7) | 49.6 (48.3, 51.0) | 24.7 (23.8, 25.5) | 50.8 (49.6, 51.9) |
| **2** | 1.53 (1.27, 1.79) | 0.88 (0.72, 1.05) | 18.4 (17.6, 19.1) | 11.7 (11.2, 12.3) | 17.0 (16.2, 17.9) | 10.3 (9.6, 10.9) | 59.2 (58.0, 60.3) | 31.6 (30.7, 32.5) | 53.6 (52.6, 54.6) |
| **3** | 1.52 (1.29, 1.75) | 0.92 (0.78, 1.07) | 16.4 (15.8, 17.1) | 11.3 (10.8, 11.8) | 12.7 (12.0, 13.4) | 9.0  (8.4, 9.6) | 66.6 (65.6, 67.6) | 39.2 (38.3, 40.0) | 59.4 (58.4, 60.4) |
| **4** | 1.60 (1.39, 1.81) | 1.09 (0.94, 1.24) | 14.6 (14.0, 15.2) | 10.8 (10.3, 11.3) | 11.3 (10.6, 12.0) | 9.0  (8.4, 9.6) | 70.2 (69.2, 71.3) | 46.0 (45.0, 46.9) | 66.0 (64.9, 67.0) |
| **5 (highest)** | 1.53 (1.33, 1.73) | 1.25 (1.09, 1.42) | 13.5 (12.9, 14.0) | 11.7 (11.1, 12.2) | 13.2 (12.5, 13.9) | 12.2 (11.5, 12.9) | 69.7 (68.7, 70.6) | 53.0 (51.9, 54.1) | 76.8 (75.5, 78.0) |
